# Combination of irreversible electroporation with sustained release of a synthetic membranolytic polymer for enhanced cancer cell killing

**DOI:** 10.1038/s41598-021-89661-y

**Published:** 2021-05-24

**Authors:** Samuel M. Hanson, Bruce Forsyth, Chun Wang

**Affiliations:** 1 Department of Biomedical Engineering, University of Minnesota, 7-105 Hasselmo Hall, 312 Church Street S. E., Minneapolis, MN 55455 USA; 2 Boston Scientific Corporation, Maple Grove, MN USA

**Keywords:** Cancer therapy, Cancer, Biomedical engineering, Drug delivery

## Abstract

Irreversible electroporation (IRE) is used clinically as a focal therapy to ablate solid tumors. A critical disadvantage of IRE as a monotherapy for cancer is the inability of ablating large tumors, because the electric field strength required is often too high to be safe. Previous reports indicate that cells exposed to certain cationic small molecules and surfactants are more vulnerable to IRE at lower electric field strengths. However, low-molecular-weight IRE sensitizers may suffer from suboptimal bioavailability due to poor stability and a lack of control over spatiotemporal accumulation in the tumor tissue. Here, we show that a synthetic membranolytic polymer, poly(6-aminohexyl methacrylate) (PAHM), synergizes with IRE to achieve enhanced cancer cell killing. The enhanced efficacy of the combination therapy is attributed to PAHM-mediated sensitization of cancer cells to IRE and to the direct cell killing by PAHM through membrane lysis. We further demonstrate sustained release of PAHM from embolic beads over 1 week in physiological medium. Taken together, combining IRE and a synthetic macromolecular sensitizer with intrinsic membranolytic activity and sustained bioavailability may present new therapeutic opportunities for a wide range of solid tumors.

## Introduction

Electroporation is the process of delivering a series of short electrical pulses to create tiny defects or “pores” within a cell membrane. If theses pores are transient and the cell membrane is able to recover, the process is referred to as reversible electroporation (RE). On the other hand, irreversible electroporation (IRE) involves the use of high-voltage short electrical pulses to create permanent pores within a cell membrane, leading to cell death by membrane lysis or loss of homeostasis^1^. While a common goal of RE is to increase the uptake of membrane-impermeable entities while minimizing cell injury^2^, Davalos et al. in 2005 proposed to use IRE as a way to kill cancer cells^3^.

IRE is used clinically as a focal therapy to ablate tumors in the prostate, liver, pancreas, and kidneys^4–6^. In contrast to thermal ablation modalities that rely on extreme heating or cooling^7^, IRE can be applied safely near large blood vessels or vital tissue structures^8^. It also spares the extracellular matrix, allowing for faster healing of healthy tissue while minimizing scarring^9^. Despite many advantages, IRE is usually considered a “last resort” for patients who do not respond to, or are not candidates for, other therapies^10^. A critical disadvantage of IRE is the inability to ablate large tumors (e.g. > 3 cm in diameter) with an electric field strength that is safe to the patient^11^. IRE relies on two or more needle electrodes to deliver electric pulses. When the electric field intensity decreases sharply with the distance from an electrode^12^ to the point below the effective threshold for IRE (500–1000 V/cm, depending on the cell type^1^), the cancer cells will only undergo RE and remain viable, resulting in incomplete tumor ablation^13^. While it is possible to increase the ablation volume by applying high voltages, doing so carries the risk of damaging adjacent healthy tissue^14,15^ and generate excessive heat near the electrodes due to Joule heating^16^. Repeated IRE treatments can be performed, attempting to fully ablate a tumor, but this is impractical under most clinical settings^4,6^.

Lowering the electric field threshold for cancer cell killing is an appealing approach to achieving large tumor ablation volume without using dangerously high voltage. Numerous reports show that cells treated with cationic molecules (such as procaine, tetracaine, lidocaine, and polyarginine), sodium dodecyl sulfate (SDS, a small-molecule surfactant) and dimethyl sulfoxide (DMSO, a polar aprotic solvent) can be killed by IRE at moderate electric field strengths^1,11,17–19^. The explanation of this effect is that the cations interact electrostatically with the anionic cell membrane, making it easier for IRE-induced pores to form^17,19^, whereas surfactants and DMSO interact with membrane lipids to alter the membrane’s edge line and surface tension, making it more difficult for pores to reseal^11^. It is important to note that with the exception of polyarginine, none of these IRE sensitizers are cytotoxic themselves.

**Figure 1:**
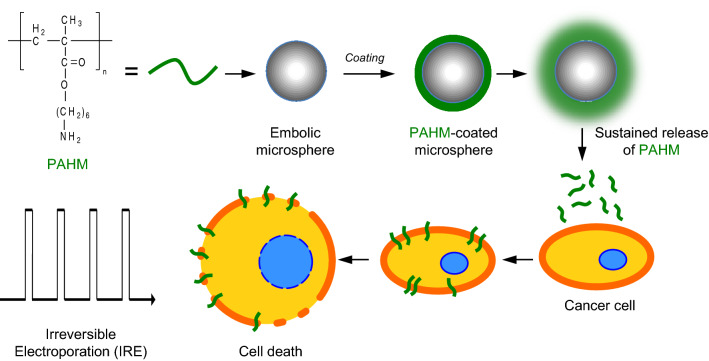
Schematic illustration of the combined killing of cancer cells by a synthetic membranolytic polymer (PAHM) and IRE. PAHM is coated onto embolic microspheres and then released to interact with cell membrane and sensitize the cells to IRE-induced membrane disruption and cell death.

Certain cationic peptides and synthetic polymers show membranolytic activity towards mammalian cells and are being investigated as anticancer agents^20–23^. Synthetic polymers with non-natural chemical structures may have advantages over peptides and polypeptides due to their chemical and biological stability, flexibility in structure, and the ease of synthesis on a large scale^21,22^. Here we postulate that a synthetic membranolytic polymer with non-natural structure, poly(6-aminohexyl methacrylate) (PAHM), will not only serve as an IRE sensitizer by lowering the electric field threshold, but also synergizes with IRE to achieve greater cell killing due to its own cytotoxicity through membrane lysis (Fig. 1). PAHM homopolymer was reported previously for its potential antimicrobial activity^24^. It lyses red blood cells, presumably due to a combination of positively charged primary amines and hydrophobic spacer arm in the side chains^25^. We further recognize the need for localized and sustained delivery of PAHM to maximize cancer cell killing while avoiding damage to healthy cells. To this end, we set out to develop a method of sustained release of PAHM from the surface of polymer microspheres (Fig. 1), which are already widely used clinically as embolic agents to treat local solid tumors^26^. In this paper, the killing of human pancreatic cancer cells by IRE and the membranolytic PAHM, applied separately or in combination, was evaluated. Embolic microspheres coated with PAHM were prepared and the release of PAHM was demonstrated. Finally, the combined effect of cancer cell killing by IRE and PAHM delivered by the embolic microspheres was analyzed.

## Results

### Tumor cell killing by IRE and PAHM applied individually

**Figure 2:**
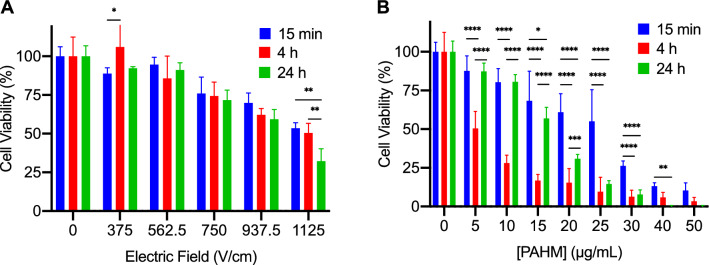
Cell viability after (**A**) IRE or (**B**) PAHM treatment applied separately. Data are shown as mean ± SD (**A**) (n = 4–6), (**B**) (n = 9–13). ANOVA with Tukey HSD test (*p < 0.05, **p < 0.01, ***p < 0.001, ****p < 0.0001).

Human pancreatic cancer cells (AsPC-1) were exposed to various doses of IRE or PAHM and cell viability was determined at various time points. As expected, higher electric field strengths led to lower cell viability (Fig. 2A). For cells exposed to 750 V/cm and below, over 70% of the cells remained viable. Even the highest electric field strength (1125 V/cm) only killed 68% of cells after 24 h. In comparison, PAHM had a more potent dose-dependent cell killing effect (Fig. 2B). Greater than 85% of cells were killed when exposed continuously to ≥ 25 µg/mL of PAHM for 24 h. With 40 or 50 µg/mL of PAHM 100% of the cells were dead after 24 h. Incubating the cells for 24 h after IRE treatment had no influence on cell viability with the exception of 1125 V/cm, which caused more cell death at 24 h than 4 h (Fig. 2A). In contrast, cell killing by PAHM increased with exposure time (Fig. 2B). Interestingly, treating cells with PAHM for 4 h appears to result in lower cell viability than treating cells for 24 h. At PAHM doses ≤ 20 µg/mL, there was a significant reduction in cell viability after 4 h of exposure, but the cells appeared to recover after 24 h. However, at PAHM doses ≥ 25 µg/mL, this recovery did not occur, as there was no statistically significant difference in cell viability between 4 and 24 h of exposure.

### Enhanced tumor cell killing by IRE/PAHM combinations

**Figure 3:**
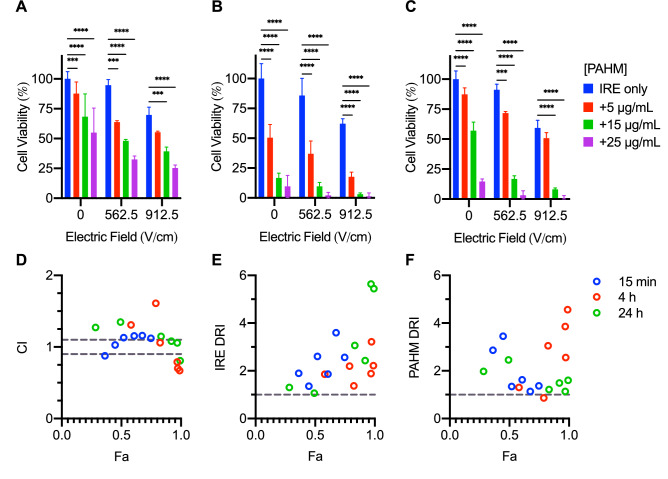
Cell viability after IRE treatment combined with PAHM exposure for (**A**) 15 min (**B**) 4 h (**C**) 24 h. Data are shown as mean ± SD (n = 3–12). ANOVA with Tukey HSD test (*p < 0.05, **p < 0.01, ***p < 0.001, ****p < 0.0001). (**D**) Combination index analysis (CI < 1, = 0.9–1 (dashed lines), > 1 indicates synergism, nearly additive effect, and antagonism, respectively). Dose reduction index analysis for (**E**) IRE and (**F**) PAHM (DRI < 1, = 1 (dashed line), > 1 indicates unfavorable dose reduction, no dose reduction, favorable dose reduction, respectively).

To evaluate the ability of PAHM to enhance the cell killing effect by IRE, two electric field strengths of IRE were combined with three PAHM doses and cell viability was assessed at various time points (Fig. 3A–C). For all exposure times, combining PAHM with IRE resulted in significantly more cell death. For example, while IRE treatment at 562.5 V/cm alone did not result in any appropriable cell death (95% viability), subsequent exposure to non-lethal doses of 5, 15, and 25 µg/mL of PAHM for 15 min reduced cell viability to 64%, 48%, and 33%, respectively (Fig. 3A). The trend remained constant and was more pronounced with 4 and 24 h of exposure to PAHM (Fig. 3B). Notably, despite cell recovery after 24 h, combined treatment of IRE at 562.5 V/cm and non-lethal doses of PAHM (5, 15, and 25 µg/mL) reduced cell viability to 72%, 17%, and 3%, respectively (Fig. 3C). Similar effect was observed with IRE at the higher 912.5 V/cm (Fig. 3A–C).

### PAHM sensitizes tumor cells for destruction by low-dose IRE

To further examine the enhancement of IRE by PAHM, the Combination Index (CI) and Dose Reduction Index (DRI) for each IRE/PAHM combination were calculated by the Chou-Talalay method^27^. The CI is plotted against the fractional affect (Fa), the fraction of cell growth inhibited by a particular combination of IRE and PAHM, calculated as [100%—cell viability in %] (Fig. 3D). For 15 min PAHM exposure, the combination of IRE and PAHM appears to be slightly antagonistic as a majority of the CI are greater than 1.12. At longer PAHM exposure, the CI tends to decrease at higher Fa. For 4 h PAHM exposure, the combinations with the three highest Fa appear to be synergistic. For 24 h PAHM exposure, combinations with lower Fa displayed slight to moderate antagonism; however, combinations with higher Fa are nearly additive or display moderate synergy. The DRI of IRE tends to increase with Fa for all three exposure times and all DRI are greater than 1 (Fig. 3E). This indicates that for any combination with PAHM, a lower electric field strength can kill an equivalent fraction of cells as a higher electric field strength alone. The DRI of PAHM for all but one combination are greater than 1 (Fig. 3F), indicating that the PAHM dose can also be reduced when used in combination. For 15 min and 24 h exposure the DRI tends to decrease with Fa; however, the DRI tends to increase with Fa for 4 h exposure to PAHM.

### Coating PAHM onto embolic microspheres

**Figure 4:**
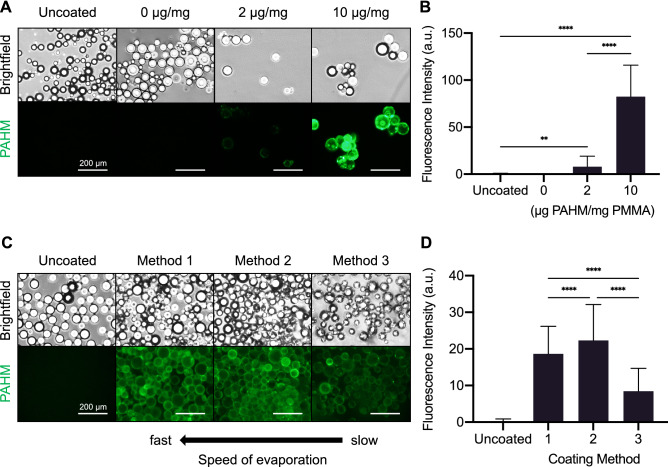
Characterization of embolic microspheres coated with fluorescently labeled PAHM. (**A**) Representative fluorescence microscopy images of microspheres coated with different amounts of PAHM (µg PAHM/mg PMMA). (**B**) Fluorescence intensity of PAHM coating on individual microspheres. (**C**) Representative fluorescence microscopy images of microspheres coated with PAHM by different solvent evaporation methods. (**D**) Fluorescence intensity of PAHM on individual microspheres after coating by different methods. Data are shown as mean ± SD (A) (n = 16–63) (**B**) (n = 165–186). ANOVA with Tukey HSD test (*p < 0.05, **p < 0.01, ***p < 0.001, ****p < 0.0001).

Embolic PMMA microspheres (100 µm in diameter) were coated with fluorescently labeled PAHM by a solvent evaporation method. Representative fluorescent micrographs (Fig. 4A) show that increasing the concentration of PAHM resulted in a thicker coating as microspheres coated with 10 µg PAHM/mg PMMA showed 10.5-fold brighter fluorescence than microspheres coated with 2 µg/mg (µg PAHM/mg PMMA) (Fig. 4B).

To optimize the coating process, embolic microspheres were coated with 4 µg PAHM/mg PMMA fluorescently labeled PAHM by three different solvent evaporation methods. Representative fluorescent micrographs of microspheres after coating are shown in Fig. 4C and quantification of the fluorescence intensity is shown in Fig. 4D. In method 1, ethanol was quickly evaporated within 10 min which resulted in a bright, relatively uniform coating (Fig. 4C). In method 2, the slower evaporation of ethanol over 12 h seemed to slightly increase the amount of PAHM coated on the microspheres, as indicated by the 1.2-fold brighter fluorescence as compared to method 1 (Fig. 4D). However, further slowing the evaporation to 3 days (method 3) resulted in a much dimmer coating (Fig. 4C,D). We chose to use method 1 to coat microspheres for all subsequent experiments because it was fast while still generating a good coating.

### Sustained release of PAHM from microspheres

**Figure 5:**
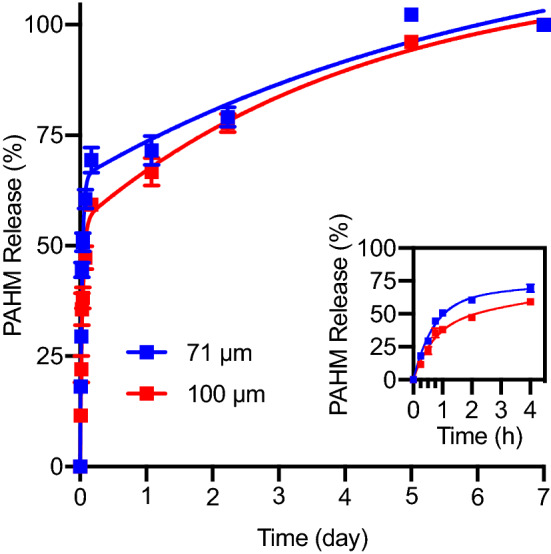
Release kinetics of PAHM from coated embolic microspheres in cell culture medium at 37 °C. Data are shown as mean ± SD (n = 4) and are fit with a two-phase exponential association model.

Microspheres (71 µm or 100 µm in diameter) coated with 10 µg PAHM/mg PMMA were submerged in cell culture medium to evaluate the release kinetics of coated PAHM (Fig. 5). PAHM was released slightly faster from 71 µm PMMA microspheres than 100 µm. After an initial burst within the first 4 h, 69% and 59% of coated PAHM was released from 71 µm and 100 µm PMMA microspheres. Subsequently a sustained release of over 20% of coated PAHM continued over the course of 1 week (between days 2 and 7).

### Tumor cell killing by PAHM released from microspheres

**Figure 6:**
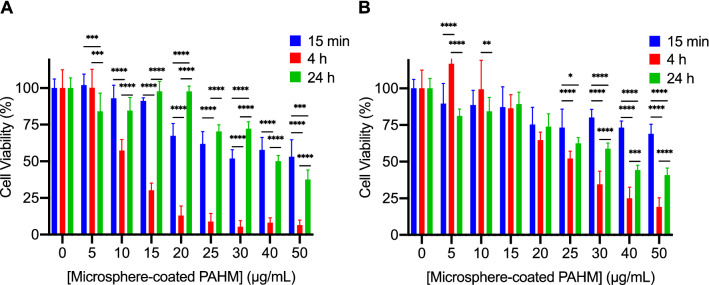
Cell viability after exposure to PAHM released from coated microspheres with average diameter (**A**) 71 µm (**B**) 100 µm. Data are shown as mean ± SD (n = 6–12), ANOVA with Tukey HSD test (*p < 0.05, **p < 0.01, ***p < 0.001, ****p < 0.0001).

The viability of AsPC-1 cells after exposure to microspheres coated with 10 µg PAHM/mg PMMA is shown in Fig. 6. PMMA microspheres alone show little to no toxicity (Fig. S1). Similar to free PAHM, the exposure time to PAHM released from 71 µm microspheres had a significant effect on cell viability (Fig. 6A). Fifteen minutes of exposure to the highest dose (50 µg/mL) of microsphere-coated PAHM killed 47% of cells. Exposure for 4 h, however, greatly reduced cell viability—less than 13% of the cells were alive after treatment with ≥ 20 µg/mL microsphere-coated PAHM. The cells recovered much of their viability after 24 h, thus only the highest dose (50 µg/mL) of microsphere-coated PAHM achieved more cell killing than at shorter time frames. Similar dose-dependent cell killing was observed for 100 µm microspheres, although the time dependence of killing was not as prominent as compared to 71 µm microspheres (Fig. 6B).

### Time and dose-dependence of cell killing by combination of PAHM-coated microspheres and IRE

**Figure 7:**
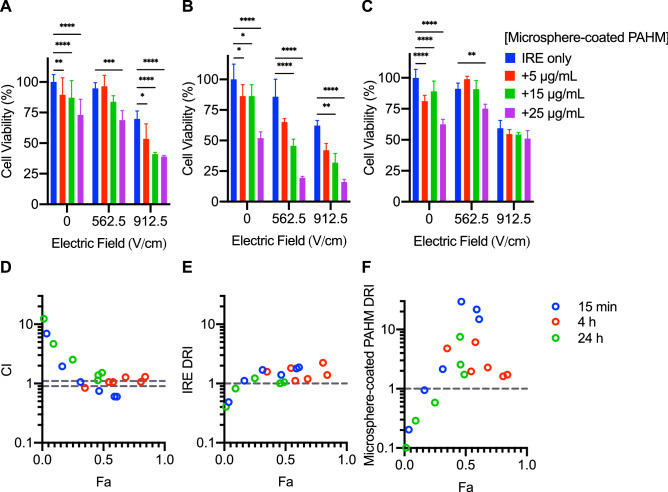
Cell viability after IRE treatment combined with exposure to PAHM released from coated embolic microspheres for (**A**) 15 min (**B**) 4 h (**C**) 24 h. Data are shown as mean ± SD (n = 3–9). ANOVA with Tukey HSD test (*p < 0.05, **p < 0.01, ***p < 0.001, ****p < 0.0001). (**D**) Combination index analysis (CI < 1, = 0.9–1 (dashed lines), > 1 indicates synergism, nearly additive effect, and antagonism, respectively). Dose reduction index analysis for (**E**) IRE and (**F**) microsphere-coated PAHM (DRI < 1, = 1 (dashed line), > 1 indicates unfavorable dose reduction, no dose reduction, favorable dose reduction, respectively).

Due to the slower release of PAHM and the much smaller differences in cell viability between 4 and 24 h exposure times, 100 µm microspheres were chosen over 71 µm ones to examine the ability of PAHM-coated microspheres to enhance IRE (Fig. 7). Released PAHM did enhance the cell killing effect of IRE after 15 min and especially 4 h, but did not achieve significant enhancement after 24 h. For 15 min exposure, the viability of cells exposed to 912.5 V/cm decreased from 95% down to 40–54% when coated microspheres were added; however, there were no statistically significant differences among the three microsphere doses (Fig. 7A). The most substantial IRE enhancement was seen at 4 h exposure to PAHM-coated microspheres, as the viability of cells exposed to 562.5 V/cm was reduced from 86 to 65%, 56%, and 19% for 5 µg/mL, 15 µg/mL, and 25 µg/mL microsphere-coated PAHM (Fig. 7B). This enhancement largely disappeared after 24 h exposure with the exception of combining 562.5 V/cm of IRE and 25 µg/mL of PAHM, which reduced cell viability to 75% from 91% by IRE alone (Fig. 7C).

### Dose reduction by combining PAHM-coated microspheres with IRE

Calculation of CI shows that combining IRE with exposure to PAHM-coated microspheres for 15 min is antagonistic at low Fa, but the combination becomes synergistic at intermediate Fa (Fig. 7D). With 4 h exposure, most of the CI are within (or close to) the 0.9–1.1 range which indicates a nearly additive effect. All combinations with 24 h exposure displayed CI greater than 1.1. At low Fa the combinations are antagonistic but tend to become additive or synergistic at medium and high Fa. The DRI for IRE tend to increase with Fa and for Fa > 0.1, the DRI for IRE is greater than 1 for all three exposure times (Fig. 7E). The DRI for microsphere-coated PAHM also trends upward with Fa with values greater than 1 for Fa > 0.2 (Fig. 7F).

## Discussion

In this study, we have shown that a synthetic membranolytic polymer, PAHM, can enhance the ability of IRE to kill human pancreatic cancer cells, and that combining IRE with PAHM reduces the doses of both IRE and PAHM needed for effecting cell killing (Figs. 2 and 3). We speculate that the mechanism of PAHM sensitizing cell membranes to IRE resembles those of cations^17,19^, surfactants and DMSO^11^. With cationic side chain, PAHM may electrostatically interact with anionic cell membrane to alter the transmembrane potential and make the membrane more susceptible to form pores at low electric field strengths^19^. Furthermore, the amphipathic properties of PAHM may make it more difficult for the IRE-induced membrane pores to heal^11^. PAHM has excellent solubility (at least 0.1 mg/mL) at mildly acidic pH (such as 6–6.5), suggesting the potential of tumor cell killing and IRE sensitization despite the hypoxic microenvironment of typical solid tumors. With the capacity of sensitizing tumor cells to low-field-strength IRE, increased volume of tumor ablation could potentially be achieved through combined use of PAHM and IRE.

The effect of cell killing by low doses (≤ 20 µg/mL) of PAHM (alone and in combination with IRE) is dependent on the duration of treatment. Unexpectedly, cell viability measured by the MTT assay was higher at 24 h than 4 h despite continued exposure to PAHM or combination with IRE (Figs. 2B, 3A–C). The MTT assay measures cellular metabolic activity as a proxy for cell viability^28^. If the cell membrane damage due to low doses of PAHM is not too extensive, the cells will be able to repair the membrane, which can take tens of minutes. During this time the cells may become temporarily less metabolically active^28^. The leaking of ATP from the damaged cell membrane can further reduce cell metabolism^29^. These phenomena may account for the apparent higher degree of cell death at 4 h. After 24 h, cells that survive PAHM treatment will have likely recovered full metabolic activity. Interestingly, such time-dependence of cell viability was not observed for IRE treatment alone (Fig. 2A). The unique and disparate temporal dynamics of IRE and PAHM treatments can be exploited further (for example, continuous exposure of low-dose PAHM accompanied by multiple, properly timed pulses of IRE), in order to achieve even greater therapeutic benefit.

A potential limitation of PAHM is its nonspecific cytotoxicity. To avoid systemic dissemination, PAHM was coated onto embolic microspheres to allow for targeted delivery to a tumor. Embolization is an established cancer therapy, in which local blood vessels are deliberately occluded to starve a tumor of its blood supply^26^. Embolic agents can be delivered simultaneously with chemotherapeutics via catheters in a process known as transcatheter arterial chemoembolization (TACE), currently under preclinical investigation^30–32^. Analogous to TACE, our PAHM-coated microspheres could also be delivered via catheter infusion to occlude tumor blood vessels and concentrate the PAHM within the tumor. The PAHM would slowly elute from the coated microspheres to accumulate locally within a tumor to sensitize the cancer cells to IRE and directly killing them. We chose to use PMMA microspheres over other types of embolic agents, such as liquids or metallic coils^26^, because conductive materials have been shown to distort the ablation zone during IRE^33^. PMMA microspheres can be produced with consistent and well-defined diameters (Fig. S2), allowing them to embolize deep, distal microvasculature of the tumors^34^. Therefore, we envision a “triple threat” strategy, where tumors can be attacked by embolic microspheres, which also release cytotoxic PAHM, which kills tumor cells either directly or through sensitization of IRE. The mutual enhancement among different therapeutic modalities and the in vivo antitumor efficacy of this strategy will be the subject of future investigation.

We developed a simple, effective method of coating PAHM onto PMMA microspheres by controlling the rate of solvent evaporation^35,36^ (Fig. 4). When submerged in cell culture medium, PAHM releases from coated microspheres over the course of 1 week (Fig. 5). PAHM releases slightly faster from 71 µm microspheres than 100 µm microspheres, presumably due to the greater surface area of the smaller particles. An initial burst release of approximately 70% of the total PAHM is seen over the first day, followed by a more sustained release in the next 6 days. This release profile could be attractive in vivo, where a high dose of PAHM is initially delivered for effective tumor cell killing followed by sustained release to sensitize any surviving cells for destruction by IRE. If low-burst, gradual release patterns are desirable, the composition and thickness of the microsphere coating may be manipulated to accomplish such goals.

Microspheres coated with PAHM were capable of killing tumor cells, although the effect was less prominent than free PAHM at equivalent doses (Fig. 6). This is expected because the sustained release profile dictates that only a fraction of PAHM was released from the microspheres at any given time (Fig. 5). Similarly, the higher percentage of cell killing by 71 µm microspheres at 4 h compared with 100 µm microspheres can be explained by the higher amount of PAHM released from the smaller size microspheres over the course of 4 h. Despite the low-level gradual release of PAHM from microspheres, significant enhancement of the efficacy of IRE was observed at 15-min and 4-h time points (Fig. 7A,B). Judging by the CI values, the nature of the enhancement is either synergistic or additive over a wide range of cell death rates (Fa = 0.3–1) (Fig. 7D). More importantly, the DRI for nearly all treatment combinations are greater than 1 (Fig. 7E,F). This suggests that lower doses of IRE/PAHM in combination have the same potency of killing cells as higher doses of the two modalities used separately. Dose reduction of IRE and/or PAHM may translate into clinical benefits including less toxic side effects, less cost and higher patient compliance.

Some have argued that the chemical sensitizers for IRE should not be inherently cytotoxic^11,19^. However, due to the stochastic nature of IRE, even high electric fields do not guarantee complete cell death. Local electrical heterogeneity within the tumor tissue can leave patches of live cells within the ablation zone. These live patches are unique to IRE ablation and often lead to tumor recurrence^1,12^. By using a cytotoxic sensitizer, PAHM, there is the possibility of eliminating these live patches for a more robust tumor ablation.

Certain IRE-related parameters, such as the number, duration, and frequency of pulses, can be optimized to improve the efficacy of IRE^1^; however, all IRE protocols would benefit from lowering the electric field threshold in the tumor tissue. IRE has also been shown to release tumor antigens^9,37–39^, which can be damaged by high electric field strengths^40^. Using PAHM to lower the electric field strength used during IRE, it may be possible to preserve a greater percentage of tumor antigens in their native form and generate a more robust antitumor immune response.

Taken together, our results suggest that a synthetic membranolytic polymer, PAHM, can be coated onto and released from embolic microspheres to reduce the electric field strength required for killing human pancreatic cancer cells by IRE. Future studies will focus on elucidating the mechanism of PAHM-modulated sensitization of cells to IRE. The timing, frequency, and duration of the treatment combinations could be optimized to achieve better cell killing. Efficacy of the combination therapy will need to be validated in appropriate in vivo tumor models.

## Conclusion

We have demonstrated that a synthetic membranolytic polymer, PAHM, when combined with IRE, led to enhanced killing of human pancreatic cancer cells. We have further developed a simple process of coating PAHM onto embolic microspheres, which provided for sustained release of PAHM. Nearly all tested combinations of PAHM and IRE caused cancer cell death in a dose-sparing manner, and some combinations achieved cell killing synergistically. These findings established that sustained release of PAHM from embolic microspheres have the potential to improve IRE-mediated tumor ablation.

## Materials and methods

### Synthesis of poly(6-aminohexyl methacrylate) (PAHM)

*N*-(tert-butoxycarbonyl) aminohexyl methacrylate (tBocAHM) was synthesized as described by Zhu et al.^41^. PAHM was synthesized via atom transfer radical polymerization (ATRP) of tBocAHM followed by deprotection of the tBoc side chains based on a method reported by Ji et al.^42^. The polymer was characterized using ^1^H NMR and gel permeation chromatography (GPC) as described by Ji^23^. The PAHM used in the subsequent experiments had a number-average molecular weight (M_n_) of 2.08 × 10^4^, Dispersity (Đ) of 1.26, and average degree of polymerization (DP) of 100.

### Fluorescence labeling of PAHM

PAHM was fluorescently labeled with the NHS ester of Alexa Fluor 488 dye (λ_ex_/λ_em_: 494/517 nm; extinction coefficient = 71,000 cm^−1^ M^−1^, Thermo Fisher Scientific, Waltham, MA) according to manufacturer’s protocol. Unreacted dye was removed by gel filtration.

### Preparation and visualization of PAHM-coated embolic microspheres

PMMA microspheres with average diameter of 71 µm or 100 µm were provided by Boston Scientific Corporation (Maple Grove, MN). PAHM-coated PMMA microspheres were prepared using a solvent evaporation method. To optimize the preparation protocol, a mixture of fluorescently labeled and unlabeled PAHM at 1:8 mass ratio was dissolved in ethanol at total concentration of 0.8 mg/mL. PMMA microspheres (100 mg) were added to PAHM solution (0.5 mL) and mixed at room temperature. The complete evaporation of the solvent was achieved either in 10 min (method 1), 12 h (method 2) or 3 days (method 3). Coated microspheres were further dried in vacuum before use. Fluorescent and bright-field images of the coated microspheres were acquired with an Olympus IX70 inverted fluorescence microscope equipped with an Olympus DP72 camera and X-Cite 120 Wide-Field Fluorescence Microscope Excitation Light Source (Excelitas Technologies, Waltham, MA). Fluorescently labeled PAHM was visualized using an excitation wavelength of 480 ± 50 nm and emission wavelength of 535 ± 50 nm. The fluorescence intensity of individual coated microspheres was quantified using ImageJ.

For PAHM release kinetics and cytotoxicity studies, the PMMA microspheres were coated with unlabeled PAHM at a concentration of 10 µg PAHM/mg PMMA by mixing 1 mL of PAHM solution (1 mg/mL) with 100 mg of microspheres. Solvent evaporation was done using method 1 as described above.

### In vitro release kinetics of PAHM from embolic microspheres

PAHM-coated microspheres (25 mg) were suspended in 1 mL of cell culture medium containing 10% serum without phenol red and were incubated in a humidified environment at 37 °C with 5% CO_2_ for 1 week. At particular time points, 750 µL of the supernatant was collected and replaced with fresh medium. The collected supernatant was diluted with 0.75 mL of release medium. PAHM in the supernatant was quantified by measuring UV absorbance at 245 nm using a Cary 100 UV–Vis Spectrophotometer (Agilent Technologies, Santa Clara, CA) (Fig. S3). Release kinetic profiles were expressed as the cumulative percentage released over time.

### Cell culture

Human pancreatic adenocarcinoma cell line AsPC-1 was obtained from ATCC. Cells were cultured in RPMI 1640 medium containing 2 g/L glucose, 2 mM l-glutamine, 2 g/L sodium bicarbonate, 10% heat inactivated fetal bovine serum (FBS), 100 U/mL penicillin, and 100 µg/mL streptomycin (Thermo Fisher Scientific, Waltham, MA) in a humidified environment at 37 °C with 5% CO_2_. When cells reached ~ 80% confluency, they were used immediately for cytotoxicity studies.

### IRE

AsPC-1 cells were treated with IRE as described previously^43^. Briefly, 400 µL of cell suspension was pipetted into an electroporation cuvette (BTX 45-0126, Harvard Apparatus, Holliston, MA) between the two aluminum plate electrodes (4 mm apart). The cuvette was placed in an external electric field created by an electric pulse generator (BTX ECM Square Wave Electroporation System, BTX Model No. 830, Harvard Apparatus, Holliston, MA), which delivered 50 electrical pulses (100 µs per pulse, 1 Hz) at 150, 225, 300, 375, or 450 V (corresponding to electric field strengths of 375, 562.5, 750, 937.5, or 1125 V/cm).

### Treatment protocols and cytotoxicity assay

Treatment with free PAHM alone: AsPC-1 cells were seeded in 12-well plates at 180,000 cells/well and grew to 80% confluency. Before treatment, all the cell culture media was removed from the wells. A PAHM stock solution of 0.1 mg/mL in cell culture media was prepared, diluted to 5–50 µg/mL with media and added to the wells at 500 µL per well. Fifteen minutes, 4 h or 24 h after treatment, the cell media was removed by vacuum aspiration. The cells were washed twice with phosphate buffered saline (PBS). Cell viability was quantified using an MTT (3-(4,5-dimethyl-thiazol-2-yl)-2,5-diphenyl tetrazolium bromide) assay^44^.

Treatment with IRE alone: AsPC-1 cells were suspended in culture media at a density of 675,000 cells/mL, and 400 µL of the cell suspension was exposed to pulsed IRE at 150, 225, 300, 375, or 450 V. The treated cell suspension was mixed with 600 µL of fresh media, transferred to 12-well plates, and cultured in a humidified environment at 37 ºC with 5% CO_2_. Fifteen minutes, 4 h or 24 h later, the cells were washed with PBS twice before the MTT assay was performed.

Treatment with combinations of PAHM and IRE: AsPC-1 cells were suspended in culture media at a density of 675,000 cells/mL. Free or microsphere-coated PAHM was added to the cell suspension at 0–50 µg/mL. After 15 min the cell suspension was exposed to pulsed IRE at various voltages. The IRE treated cell suspension was transferred to 12-well plates and cultured for an additional 15 min, 4 h or 24 h in the presence of 0–50 µg/mL of free or microsphere-coated PAHM before the MTT assay was performed.

### COMPUSYN analysis of treatment combinations

Combination index (CI) and dose reduction index (DRI) for each treatment combination were calculated by the Chou-Talalay method using the CompuSyn software program (ComboSyn Inc., Paramus, NJ)^27^. CI value is dimensionless quantification of drug interaction. CI = 1 indicates an additive effect; CI < 1 indicates a synergistic effect; CI > 1 indicates an antagonistic effect. DRI is a measure of how much the dose of each drug can be reduced if used in combination at a given fraction of cell death (Fa) as compared to the dose of each drug alone, DRI = 1 indicates no dose reduction; DRI > 1 indicates favorable dose reduction, DRI < 1 indicates unfavorable dose reduction.

### Statistical analysis

ANOVA and Tukey HSD test for multiple comparisons were used to determine the significance of difference in cell viability between different treatment groups. The same tests were used to determine the significance of difference in fluorescence intensity of PAHM on coated microspheres. PAHM release profiles were fit with a two-phase exponential association model. All analyses were performed using GraphPad Prism, version 9.0.0 (GraphPad software Inc., San Diego, California, USA).

## Supplementary Information

Supplementary Information.
