# Supplementary material for: Combination of irreversible electroporation with sustained release of a synthetic membranolytic polymer for enhanced cancer cell killing
Source: Sci Rep. 2021 May 24;11:10810. doi: 10.1038/s41598-021-89661-y (PMC8144369; doi:10.1038/s41598-021-89661-y)
Supplement: Supplementary file 1 — Supplementary Information. [file 41598_2021_89661_MOESM1_ESM.pdf]

Combination of Irreversible Electroporation with Sustained Release of a Synthetic Membranolytic Polymer for Enhanced Cancer Cell Killing

Samuel M. Hanson[1], Bruce Forsyth[2], Chun Wang[1]*

[1]Department of Biomedical Engineering, University of Minnesota, 7-105 Hasselmo Hall, 312 Church Street S. E., Minneapolis, MN 55455, USA

[2]Boston Scientific Corporation, Maple Grove, MN, USA

*Corresponding author. Telephone: 612-626-3990; Fax: 612-626-6583; E-mail: wangx504@umn.edu

Supporting Information

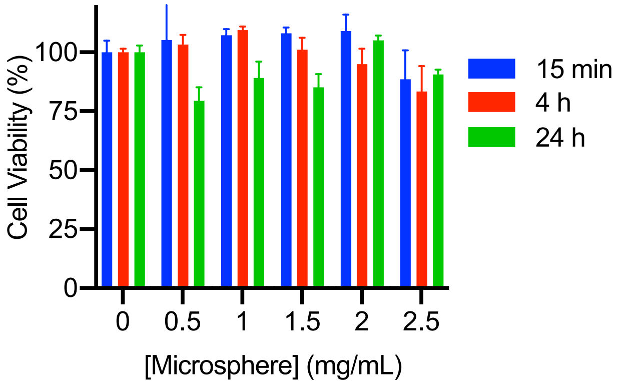

Figure S1. Cell viability after exposure to uncoated embolic microspheres. Data are shown as mean +/- SD (n = 2).

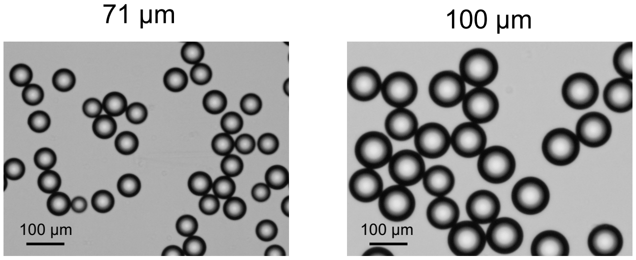

Figure S2. Representative microscopy images of uncoated embolic microspheres with average diameter 71 μm (left) or 100 μm (right)

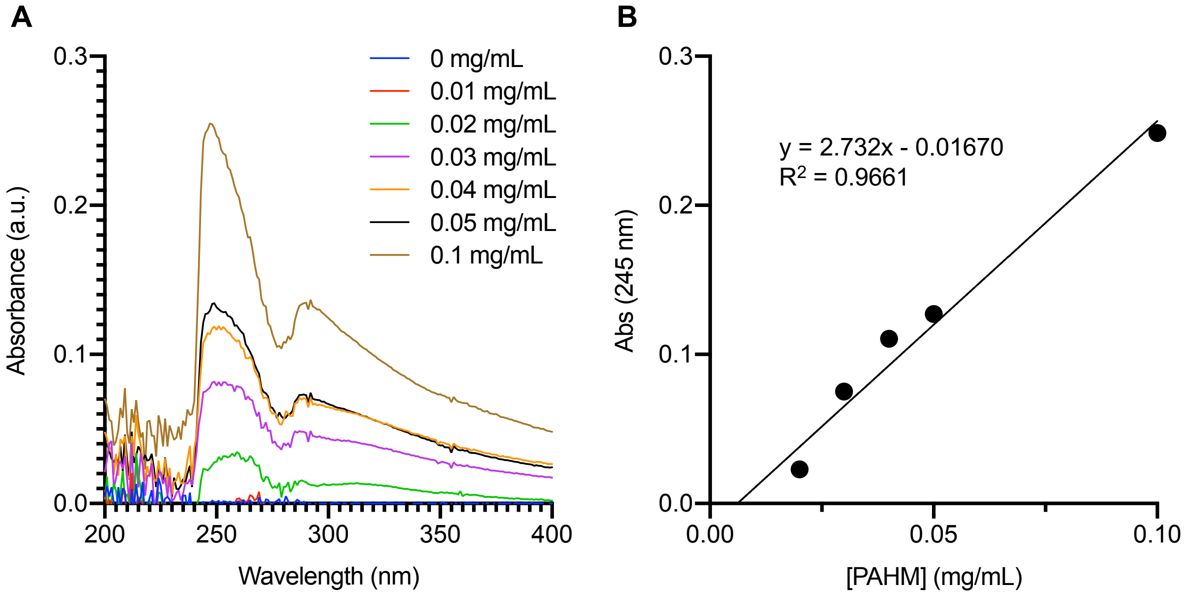

Figure S3. Quantification of PAHM in cell culture medium. (A) UV-Vis absorption spectra of aqueous PAHM solution of various polymer concentrations. (B) Standard curve showing absorbance at 245 nm vs. PAHM concentration.
